# Estrogen versus bone marrow mesenchymal stromal cells in the regeneration of the parotid gland in ovariectomized rats

**DOI:** 10.1590/1678-7757-2025-0374

**Published:** 2025-09-22

**Authors:** Sally Hassan Abo BAKER, Amira Ahmed R. MOAWAD

**Affiliations:** 1 Ha’il University College of Dentistry Department of Basic Dental & Medical Sciences Ha’il Province Saudi Arabia Ha’il University, College of Dentistry, Department of Basic Dental & Medical Sciences, Ha’il Province, Saudi Arabia.; 2 Majmaah University College of Dentistry Department of Maxillofacial Surgery and Diagnostic Science Riyadh Saudi Arabia Majmaah University, College of Dentistry, Department of Maxillofacial Surgery and Diagnostic Science, Al-Majmaah 11952, Riyadh, Saudi Arabia.; 3 Mansoura University Faculty of Dentistry Department of Oral Biology Egypt Mansoura University, Faculty of Dentistry, Department of Oral Biology, Egypt.

**Keywords:** Caspase 3, Estrogen, Menopause, BM-MSCs, Ovariectomy

## Abstract

Menopause causes specific hormonal alterations in women that make them more susceptible to salivary changes, such as decreased saliva flow, which can lead to xerostomia, modified taste, and a burning sensation in the mouth.

**Objectives:**

This study aimed to determine which of two treatments—bone marrow-derived mesenchymal stromal cells (BM-MSCs) or estrogen—more effectively ameliorate the postmenopausal degenerative effects on the parotid salivary glands of ovariectomized rats by histological, immunohistochemical, and malondialdehyde (MDA) evaluations.

**Methodology:**

The study specimen involved 70 female albino rats. Group I received saline, and were subdivided into sham-operated and vehicle-treated. Group II was subjected to bilateral ovariectomy and received no treatment. Group III was subjected to bilateral ovariectomy and, one week later, treatment with subcutaneous injections of 1 mg/kg/daily estrogen for 12 weeks. Group IV underwent bilateral ovariectomy and, one week later, received a single intraglandular injection of a BM-MSC solution. At the end of the experiment, the rats were euthanized, and their parotid glands were dissected and processed for H&E stain, caspase 3, and MDA evaluations.

**Results:**

The histological and immunohistochemical findings in Group II showed marked degenerative changes in the parotid gland. However, in Groups III and IV, regeneration was observed following treatment with estrogen and BM-MSCs, respectively. The estrogen and BM-MSCs groups showed significant decrease in MDA levels in the parotid gland relative to the ovariectomized group and nearly comparable to control.

**Conclusion:**

BM-MSCs and estrogen show histological efficacy in regenerating the parotid salivary glands of ovariectomized rats.

## Introduction

Menopause (primarily resulting from aging in women) marks the cessation of menstruation due to hypoestrogenism. These hormonal changes are significant not only for reproductive capacity but also for their broader metabolic implications.^1^ Estrogen plays a vital role in various mammalian tissues, influencing processes such as mitosis, proliferation, and growth. It also notably affects human embryogenesis and the maintenance of life.^2^

Estrogen insufficiency is interrelated to an increased hazard of developing cardiovascular diseases,^3^ metabolic bone disease, and salivary gland diseases.^4^ In rodents, salpingo-oophorectomy more greatly deteriorates serum estradiol levels,^5^ thus being comparable to a normal state of menopause and providing a significant research instrument to simulate the postmenopausal hormonal states in humans.^6^ Moreover, estrogen deficiency is also coupled with oral and salivary gland diseases, including diminution of secretion and alteration of saliva composition.^7^

Furthermore, menopause is connected to an increase in oxidative stress,^8^ implying that estrogens may have antioxidant properties. Significant decreases in estrogen have been shown to increase levels of oxidative stress in the body depending on its concentration and chemical structure. Particularly at high concentrations estrogen tends to have a useful antioxidant effect by inhibiting the 8-hydroxylation of guanine DNA bases. Although at low concentrations this hormone has pro-oxidant-like effects, especially when its chemical structure contains a catechol. These effects include breaks in genetic material, formation of DNA adducts, and oxidation of bases.^9^ Moreover, serum concentrations of inflammatory cytokines and pro-oxidant biomarkers such as glutathione, 4-hydroxynenal, and malonaldehyde were found to be higher in postmenopausal women than in premenopausal women.^10^

Salivary glands are essential oral structures that play a key role in salivation. They consist of three major glands: the parotid, submandibular, and sublingual glands, which collectively secrete approximately 95% of saliva, whereas the minor salivary glands contribute the remaining 5%.^11^ The parotid gland acini comprise only serous cells, whereas those of the submandibular gland are serous and mucous cells. The sublingual gland only consists of mucous cells.^12^ Drooling is monitored by the parasympathetic and sympathetic nervous systems.^11^ The craniosacral system releases water and ions while the thoracolumbar system triggers protein production.^13^ Furthermore, fluctuations in estrogen levels are reflected in the composition of salivary proteins and inorganic components,^14^ which, in turn, play a pivotal role in the regulation of salivary gland function.^15^

The multipotent bone marrow stromal cells (BMSCs) can differentiate into many cell types, including bone, fat, cartilage, muscle, and skin. Moreover, they have self-renewal capacity and can be derived from multiple organs, including the medulla ossium, synovia, dental pulp, birth cord, cord blood, Wharton’s jelly, corneal cells, body fats, placenta, and fetal fluid.^16^ MSCs have greater practical potential due to their biologically active properties: (i) the capacity to reach inflammatory sites of damaged tissues when intravenously injected, (ii) the capability of differentiation into many cell types (such as adipocytes, chondrocytes, myocytes, osteoblasts, and connective tissue cells), (iii) the release of many biologically active substances (thus enhancing the buildup of damaged cells), and (iv) their activity as anti-inflammatory agents and immune system modulator functions.^17^

BMSCs have been used in cytotherapy for various diseases and have shown beneficial effects in clinical and preclinical studies.^18^ As of January 3, 2022, more than 1,500 clinical trials involving mesenchymal stem/stromal cells are registered on ClinicalTrials.gov. Due to their multipotent nature, BMSCs are expected to contribute to tissue regeneration by differentiating into specific cell types. Nevertheless, available evidence suggests that BMSCs exert their therapeutic effects primarily via paracrine mechanisms, by releasing growth factors, cytokines, and extracellular vesicles.^19,20^

BMSCs show promise in the treatment of many illnesses, including myopathy, osteopathy, neuropathy, osteoarthritis, hematological diseases, attitudinal and mental health disorders, autoimmune diseases, viral or communicable diseases, tumors, wounds, and tissue damage.^21^

Since estrogen therapy enhances women’s life satisfaction,^22^ it is commonly used to manage the vasomotor symptoms of menopause. However, high doses of estrogen are associated with an increased risk of endometrial and breast cancers.^23^ This has prompted researchers to explore alternative options to estrogen or to develop multimodal therapies that can mitigate the risks associated with estrogen monotherapy.

Therefore, this research aimed to compare the restorative efficacy of estrogen administration with that of injections of bone marrow mesenchymal stromal cells in ameliorating the menopausal symptoms manifested in the parotid gland in an albino rat model.

## Methodology

### Animals

In total, 74 adult female albino rats aged four months and weighing 200–250 grams were used in this study. The animals were housed in individual cages, provided with a balanced diet, and maintained under secure and appropriate conditions.

After one week of acclimatization, 10 rats were used for the isolation of BM-MSCs. Moreover, 64 rats were randomly divided into four groups each:

Group I (control group): 16 rats that were subdivided into two subgroups: Ca (sham-operated) and Cb (vehicle-treated).

Group II: 16 rats were subjected to bilateral ovariectomy but no treatment.

Group III: 16 rats were subjected to bilateral ovariectomy; one week after they became ovariectomized (OVX), the rats were given subcutaneous injections of estrogen (17-estradiol-water soluble, 1 mg/kg/daily for 12 weeks. Estrogen occurs as estradiol benzoate; an active ingredient under the name FOLONE in the ampoules of the oily solution (Misr company for pharmaceuticals).^24^

Group IV: 16 rats were subjected to bilateral ovariectomy; one week after they became OVX, they were treated by intraglandular injection of isolated BM-MSCs solution once. (Each rat received a 106 BM-MSC-suspension per 250 gm of body weight).^25^

Sample size: 74

N value: 16

The sample size and N value were calculated according to certain factors such as the acceptable level of significance (P value), study power (1 − β), the expected ‘clinically relevant’ effect size, and underlying event rate in the population.

### Histological evaluation

At the end of the experiment, the rats were euthanized with an overdose of halothane. Their parotid glands were promptly excised and placed in 4% buffered paraformaldehyde (pH 7.4) for 12 h. The tissues were then dehydrated with a series of graded ethyl alcohol and embedded in paraffin. The blocks were cut into 6-7-µm sections using a motorized rotary microtome (RMC Products, Tucson, AZ, USA). For the histopathological analysis, sections of each sample were stained with hematoxylin and eosin (H&E), and for the histochemical analysis, with caspase 3. Histochemical changes observed via microscopy were scored according to the following results: negative or none, mild, moderate, and intense.^26^

### Immunohistochemistry evaluation

Immunohistochemical staining was then performed. First, paraffin sections were dewaxed, hydrated, and then incubated in 3%H_2_O_2_ for 20 min to block endogenous peroxidase. Second, the sections were placed in goat serum for 2 h, and then incubated in rabbit anti-rat Cu-Zn SOD multiclonal antibody (1:500 dilution; Santa Cruz) and rabbit anti-rat Casp-3 multiclonal antibody (1:100 dilution; Beijing Zhongshan Golden bridge Biotechnology Co, Ltd) at 4°C for 24 h. Then, the biotinylated goat anti-rabbit IgG serum and ABC solution (Beijing Zhongshan Golden bridge Biotechnology Co, Ltd) were successively added to the sections, and each reagent was allowed to react at room temperature for 3 h. In the end, the sections were stained with a DAB kit, obtaining brown immunoreactive products. The negative control was processed in the same manner as above. However, the rabbit anti-rat Cu-Zn SOD and Casp-3 polyclonal antibody were replaced with 0.1 mol/L PBS.^27^

### MDA evaluation

The parotid gland specimens were preserved in 1ml saline solution and kept frozen at −18^0^c.^28^

### Statistical analysis

Statistical analyses were performed to determine the differences between the four groups. Data are shown as mean ± standard deviation of the mean. The data were analyzed using the Statistical Package for the Social Sciences (SPSS), version 20.0. One-way ANOVA and the post hoc test were also performed. For all statistical tests, a *P* ≤ 0.05 was considered as the level of significance.

### Bilateral ovariectomy (OVX)

Bilateral ovariectomy was performed by veterinarians of the Mansoura University animal house. The animals in groups II, III, and IV were anesthetized with ketamine by an injection in the peritoneal cavity (75 mg/kg body weight) before surgery.^24^ The lower part of their back was shaved and a single 1.5-2-cm incision was made in their skin to expose their back muscles. Then, a 2-cm incision was made in the muscles overlying the ovaries on both sides, and the ovaries were isolated, tied off with sterile suture, and removed. The muscles and the skin were sutured separately. To prevent wound damage from other animals, they were caged separately for post-surgical care.^29^ The surgical wound of the operated rats was cleaned with povidone iodine twice a day for five days.^30^ Each rat received an intramuscular injection of 0.1 ml Penicillin G procaine (300,000-unit ml-1, Phoenix Pharmaceutical Inc., St. Joseph, MO) as a preventative measure. The sham-operated group received the fake operation, the removal of a small piece of fat (the volume of fat was equal to that of the removed tissue in OVX rats), and the preservation of both ovaries.^31^ After surgery, the health status of the rats was monitored daily.

### Inclusion criteria

The selection of the animal models for the experiment was based on the following considerations: 1) usefulness as an analog, 2) transmittable information, 3) organism genetic homogeneity when applicable, 4) background awareness of biological properties, and 5) cost and availability.

### Exclusion criteria

Animals were excluded if impediment was expected during parotid gland excision or in case of failure to reach quality control excellence, such as unsatisfactory levels of pollutants or mediocre histological quality.

### Preparation of BMSCs

#### Isolation of mesenchymal stromal cells from rat bone marrow

In total, 10 four-month-old females were euthanized by cervical dislocation and their femurs and tibiae were meticulously cleaned from skin by pulling toward the foot, which is cut at the ankle bone. The muscle and fibrous tissue were excised from the tibia and the femur by scraping the diaphysis of the bone clean then dragging the tissue toward the bone end. The bones were put in ethyl alcohol 10% for antisepticising and left in it for just seconds. The ends of the tibia and femur were cut by sharp scissors. A 27-gauge needle was inserted and flushed with DMEM (Dulbecco’s Modified Eagle’s Medium) to be collected in a 15-ml tube. The cell suspension was filtered through a 70-μm filter mesh. The BM cells were cultured in DMEM+10% FBS+1% antibiotic-antimycotic solution (Thermo scientific, USA) in 25-cm^2^ tissue culture flask and incubated at 37°C with 5% CO_2_.^32^

## Cell culture conditions

After one day, non-compliant cells were removed by two-three washes with phosphate buffered saline (PBS). Compliant cells further cultured in complete medium. The medium was changed every three days until the monolayer of adherent cells reached 70~80% confluence. Cell passaging was performed using trypsin-EDTA solution (0.25%, Sigma Aldrich, USA). The number of recovered cells were assessed using the hemocytometer and cell survival was quantified by the trypan blue exclusion test. Approximately 250–300×103 cells were used to inoculate 75-cm2 culture flask. The cultures were incubated at 37°C and 5% CO2. Cell cultivation was performed up to the third passage.^32^

## Flow cytometry analysis

MSCs were harvested *in vitro* from the tissue culture flasks after passage 3 and centrifuged at 200g for five minutes at ambient temperature. The cells were washed and counted in a Neubauer Chamber. A single cell suspension of 0.5 to 1x 106 cells was placed in 50μL of buffer (PBS, 0.1% sodium azide, 2% FBS). The cells were incubated with primary antibody for 40 min following the direct staining method with impregnating concentrations of monoclonal antibodies CD105, CD34, and CD90.^33^

## Results

### Histological results

**Figure 1:**
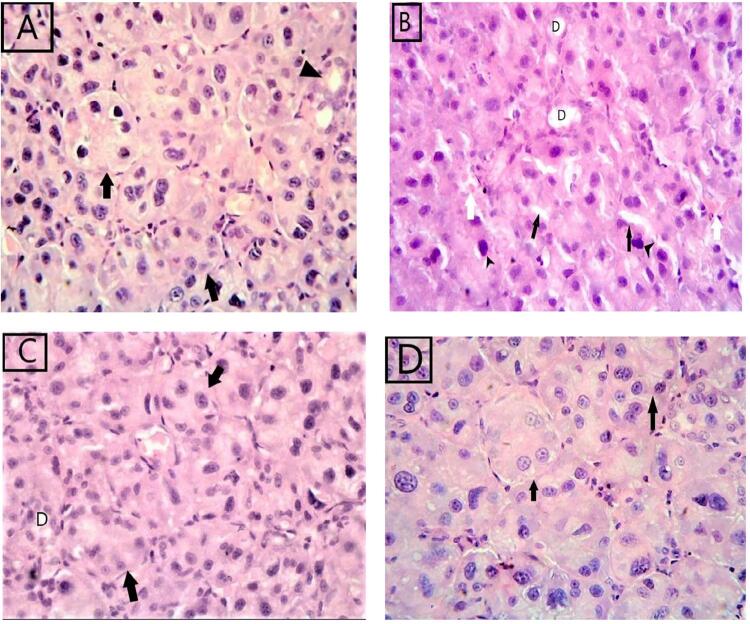
Photomicrograph of parotid gland H&E-stained sections. (H&E stain, x400). (A): Control group (group I) showing rounded serous acini with narrow luminae (arrow). They have basal basophilic nuclei and apical acidophilia. Also note normal appearance of intralobular ducts (arrow head) (B) group II showing irregular acini with darkly stained nuclei (arrow heads) and cytoplasmic vacuolations (black arrow), dilated interlobular ducts. (D) congestion of blood vessels (white arrow). (C) group III showing restoration of the normal architecture of acini (arrow), the interlobular ducts are regular with relatively wide lumina (D). (D) group IV showed marked improvement in the acinar architecture of the parotid lobules including the acini (arrow), which are arranged in groups separated by narrow interlobular septa

The examination of H&E-stained sections showed that group (I) (Ca & Cb) had the same structures. The classic architecture of the parotid gland appeared in the form of serous acini and duct systems. The acini appeared rounded with secretory cells that had large rounded basophilic nuclei. Narrow lumina and apical acidophilia were evident. The intercalated ducts formed a monolayer cubic cell with a round nucleus and normal structure. Thin connective tissues appeared around ducts, blood vessels, and in-between lobules. (Figure 1A)

The stained sections in group (II) mostly showed irregular acini with darkly stained nuclei and many vacuoles in their cytoplasm, condensation of chromatin in the nuclei, and distended and clogged blood vessels. Expanded striated and interlobular ducts were also found, and some of the epithelial lining of the ducts showed darkly stained nuclei. Degenerated cells with ruptured membrane appeared in the acini and ducts (Figure 1B).

In the stained sections of group (III), the approximately normal appearance of the serous acini and ducts were seen, but they had few vacuoles in the cytoplasm. The ducts appeared with regular walls and narrow lumina. (Figure 1C)

The histological sections of the rat parotid gland in group (IV) showed marked improvement in the parenchymal architecture of the parotid gland. The serous acini showed normal outline arranged in groups, ducts with preserved regular walls, and narrow lumina. (Figure 1D)

### Immunohistochemical results

The stained sample slides are generally evaluated under a light microscope by trained researchers. Semi-quantitative scoring systems (negative/mild/moderate/intense) are widely used to convert subject perceptions of immunohistochemistry marker expression into (semi) quantitative data, which is then used for statistical analyses and conclusions. The existing clinical scoring process is based on two characteristics: overall staining intensity and the proportion of stained tissue or cells.

**Figure 2:**
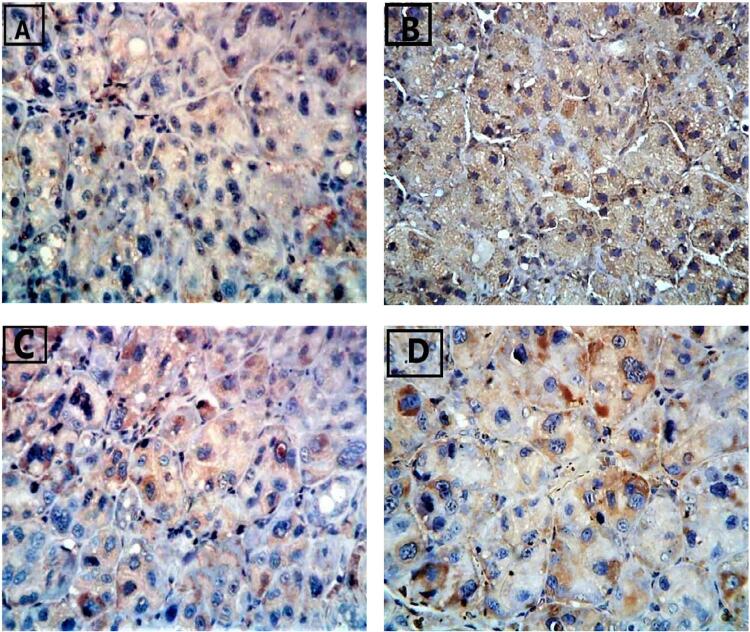
Photomicrograph of parotid gland sections (immunoperoxidase staining with anti-caspase 3 antibody, DAB chromogen X400). A: Group I with a mild reaction to caspase 3 in the cytoplasm of some acini. B: Group II with an intense reaction to caspase 3 in the acinar cells. C: Group III with a moderate reaction to caspase 3 in the cytoplasm of the acinar cells. D: Group IV with a mild reaction to the caspase 3 surrounding the nuclei of the acinar cells.

**Figure 3:**
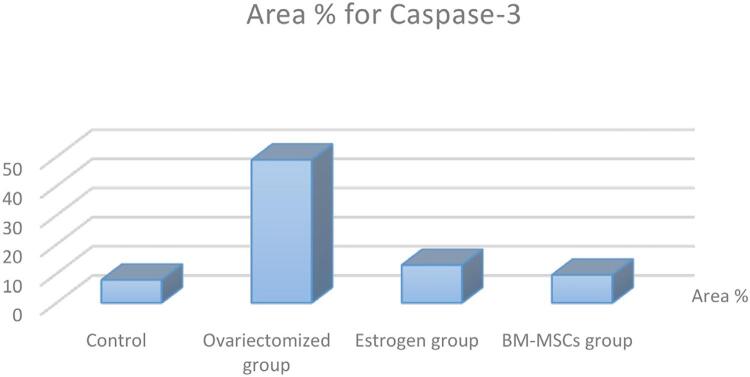
Bar chart showing the statistical analysis for caspase 3 immunostaining.

The immunohistochemical positive results were detected as brown deposits by using DAB (3,3’-diaminobenzidine) chromogen stain. The expression of caspase 3 was considered to be positive in case of any staining of the cytoplasm, regardless of staining intensity (Figures 2, 3 and Table 1).

**Table 1:** High immunoreactivity of caspase 3 in ovariectomized group compared to the control, estrogen, and BM-MSCs groups.

Caspase 3	Control	Ovariectomized group	Estrogen group	BM-MSCs group
Area %	7.89±0.27***	48.97±9.18***###	12.98±2.70***	9.57±1.76***

Data shown as mean±SD, P: Probability: significance <0. 05

Test used: One-way ANOVA followed by the Tukey’s post-hoc test

*P <0.05; **P<0.01; ***P<0.001 vs. Control group.

#P <0.05; ##P<0.01; ###P<0.001 vs. ovariectomized group.

4Group I (Control group):

The normal parotid sections showed a mild reaction to caspase 3 in the cytoplasm of some acini, Figure 2 A).

Group II:

Sections showed an intense reaction to caspase 3 in their acinar cells. (Figure 2 B).

Group III:

Sections showed a moderate reaction to caspase 3 in the cytoplasm of their acinar cells, Figure 2 C).

Group IV:

Sections showed a mild reaction to caspase 3 surrounding the nuclei of their acinar cells, Figure 2 D).

### MDA results

**Figure 4:**
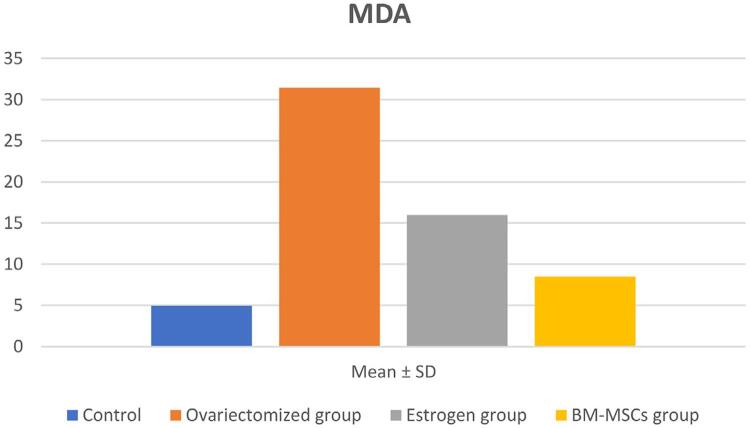
Bar chart showing the statistical analysis for MDA test.

One-way ANOVA results showed significant difference between all groups; Group II showed the highest values and group I, the lowest values regarding Mean ± SD (Figure 4 and Table 2).

**Table 2:** Higher values of oxidative stresses indicated by increased Mean ± SD of MDA in ovariectomized group when compared to the estrogen and BM-MSCs groups.

MDA	Control	Ovariectomized group	Estrogen group	BM-MSCs group
Mean ± SD	4.94±1.67***	31.44±1.79***###	15.99±2.08***	8.48±0.59***

Data expressed as mean±SD, P: Probability: significance <0. 05

Test used: One-way ANOVA followed by the Tukey’s post-hoc test*P <0.05; **P<0.01; ***P<0.001 vs. Control group.

#P <0.05; ##P<0.01; ###P<0.001 vs. ovariectomized group.

Baker SH, Moawad AA

### Cell culture of BMMSCs

MSCs attached to the culture flasks sparsely and showed a fibroblast-like, spindle-shaped morphology during the initial days of incubation. After from three to four days of incubation, proliferation started and the cells gradually grew into small colonies. By the time they were six-eight-days old, colonies with different sizes increased in number. As expansion continued, adjacent colonies interconnected with each other and a monolayer confluence was obtained after 12–15 days of incubation. In later passages, MSCs showed large, flattened, or fibroblast-like morphology and failed to change throughout 25 passages. Tests for bacterial and mycoplasma contamination were negative and its viability exceeded 95%.

### Immunophenotypic characterization

**Figure 5:**
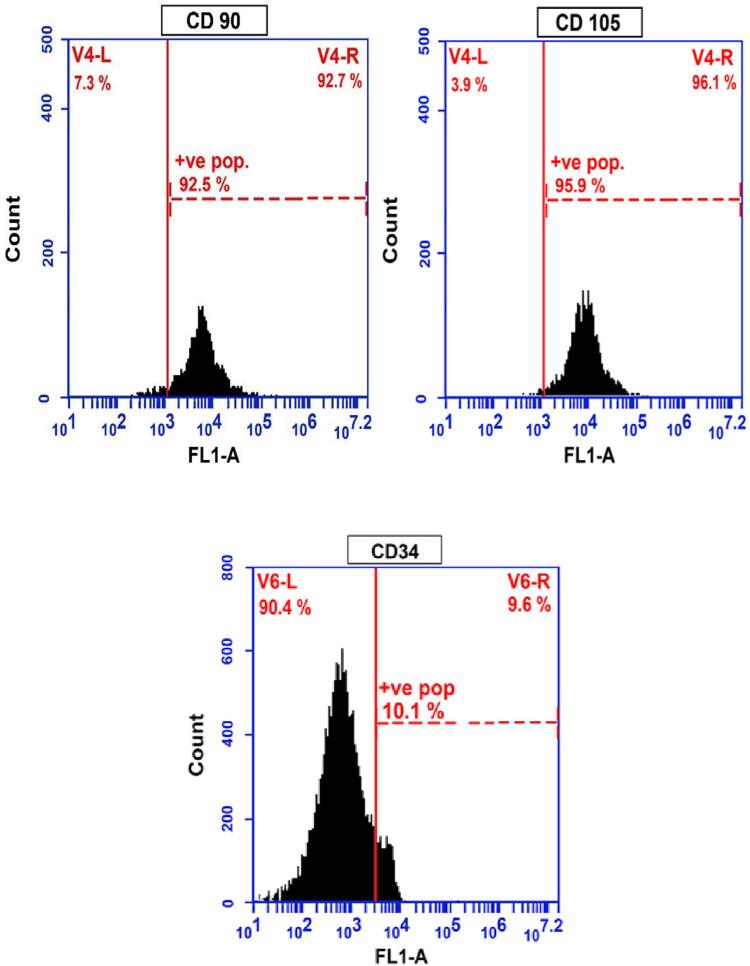
Photographs showing flow cytometry results.

Cultures of third-passage BMSCs were analyzed for expression of MSC specific cell-surface markers. BMSCs were negative for the hematopoietic lineage marker CD90 and CD105 with average percentages of 7.30±0.23 and 3.90±0.18, respectively. BMSCs were positive for CD34 with average percentages of 90.2±0.41 (Figure 5).

## Discussion

Regenerative medicine is believed to be an effective instrument for several clinical implementations.^34^ Certain surveys have shown stromal cells from bone marrow to be typical mesenchymal stem cells (MSCs). The International Society for Cellular Therapy specified the minimal criteria for mesenchymal stem cells.^35^ MSCs have been shown to differentiate into independent cell lineages that can act as immune system modulators and reduce inflammation.^36^

Menopause is characterized by a reduction in estrogen levels, which negatively affects quality of life. Insulin resistance and redox imbalance, both associated with menopause, contribute to various disorders in body systems, including the salivary glands.^37^ Ovaries are the principal source of natural estrogens. Their malfunction decreases blood estradiol levels and its stimulatory function in target tissues. The presence of estrogen receptors in soft tissue cells causes changes elicited by lack of natural estrogen.^38^

Menopause induced by surgical ovariectomy furnishes a proper way to study menopausal disorders in animal models. The results of this study provided evidence that ovariectomy induces different forms of deterioration in the parotid glands in group II. Light microscopic examination of the parotid gland sections of group II showed aggravating parotid architecture with the deterioration of acinar cells. These results agree with Mohamed, Elnegris, and Wahdan^39^ (2015), who proved that ovariectomy induces many varieties of deteriorating effects on the parotid gland. Most of the acini appeared disorganized, with darkly stained nuclei and many vacuoles in their cytoplasm; the ducts between lobules were extended and delineated by a stratified epithelium. Congested blood vessels were also found.

Moreover, as estrogen regulates the balance between endothelial-dependent vasoconstrictor and vasodilator influences [possibly via estrogen-specific actions on nitric oxide- and prostaglandin (PG2)-dependent pathways], this explained the vasodilatation of blood vessels in group II.^40^

The findings of this study also agree with those in Büyük, et al.^41^ (2015), who reported the deterioration of striated duct cells and serous acini, accompanied by pyknotic nuclei and acidophilic cytoplasm. A few polymorphonuclear cells were observed infiltrating the fibrous tissue of the parotid glands in rats 12 weeks after ovariectomy. The authors attributed these pathological changes to the absence of estrogen receptors in the salivary glands and suggested that estrogen exerts free-radical scavenging effects in these tissues. Moreover, Tirapelli, Tirapelli, and Schimming^42^ (2001) explained the presence of cytoplasmic vesicles in their group II by the great penetration of fluids in the cytoplasm of the acinar cells.

The mechanism of salivary gland dysfunction due to hormone deficiency, including menopause, remains unclear. Lipid deposition and excessive production of reactive oxygen species induce dysfunction in the salivary glands of female rats. Recently, it was reported that xerostomia caused by salivary gland dysfunction after menopause results from the ferroptosis caused by increased lipid peroxidation and iron accumulation in the submandibular gland. Lipid deposition and changes in the acinar and ductal cells, causing the estrogen deficiency in this study, are also associated with ferroptosis.^43^

In this study, after administering estrogen in group III, the serous acini and ducts can be seen as having a relatively normal architecture with minimal cytoplasm vacuoles. These findings agree with Leimola-Virtanen, et al.^44^ (2000), who stated that female sex steroids may positively impact salivary glands. It is also worth noting that the prevention of postmenopausal hypoestrogenism can be based not only on simple supplementation with the missing hormone, but also by drugs modulating estrogen receptors, e.g., raloxifene.^45^ It has been mentioned that a tissue-specific estrogen receptor is widely expressed in oral epithelia and the salivary glands. Consequently, estrogen appears to play an important and intricate role in the regulation of salivary glands, especially of ductal cells.^46^

Also, Mohamed, Elnegris, and Wahdan^39^ (2015) described that the examination of ovariectomized rats after treatment with estrogen clearly showed the normal architecture of serous acini and ducts with a moderate amount of collagen fibers.

In this study, the flow cytometric analysis of expressed surface antigens was used, which is consistent, fast, and dependable and can characterize MSCs despite whether trypsin was used to remove the cells from the substrate.^47^ MSCs have been reported to be uniformly positive for CD34 and negative for CD105 and CD90.

The histological results of the rat parotid gland in stem cell group IV showed a significant enhancement in parotid parenchymal architecture, with the normal appearance of the serous acini. These findings agree with those in Denewar and Amin^48^ (2020), who reported a more regular glandular architecture of the parotid gland following intravenous injections of bone marrow-derived stromal cells in diabetic rats. Fotino, et al.^49^ (2010) also stated that tissue repair and regeneration by BMSC transplantation is achieved by stimulating immune mechanisms.

Caspase-3 is a frequently triggered death protease that is significant for cell death in a conspicuous tissue-, cell type- or death stimulus-specific manner, and is necessary for some of the distinctive changes in cytomorphology and specific biochemical events interrelated with the performance and completion of apoptosis.^50^

In this study, glands of the control group stained with anti-caspase 3 showed a mild reaction in the cytoplasm of some acini, whereas group II, a severe reaction. Results for group III showed a moderate reaction of anti-caspase 3 in the cytoplasm of the acinar cells in comparison to group II, indicating a reduction in apoptosis. Moreover, group IV showed a mild reaction to the caspase 3 surrounding the nuclei of the acinar cells. It is noteworthy that the acinar and ductal cells in group II had condensed polymorphic or pyknotic nuclei, which are presumed to be characteristic of apoptotic cells. These detections agree with Moawad, et al.^51^ (2016), who proved that anti-caspase 3 showed a mild reaction in the cytoplasm of some acini of the parotid gland in the control group, a reaction which may be due to the fact that apoptosis permits the body to kill and remove undesirable cells during animal development, normal equilibrium, and disease.

The findings in this study are further supported by Monroe, Berger, and Sanders^52^ (2002), who have shown that estrogen plays a critical role in protecting against apoptosis in various cell types. They reported that estrogen deficiency increases tissue apoptosis in organs such as the brain, endothelia, the testes, and uterus. Furthermore, Kiray, et al.^53^ (2007) reported that oxidative stress caused by estrogen deficiency in group II is the major factor that induced cellular damage, which might trigger apoptosis.

Further, the results in this study are also compatible with Monroe, Jin, and Sanders^54^ (2000), who proved that estrogen withdrawal activates several caspases. They also showed that the BMP-7 gene is transcriptionally upregulated following estrogen depletion and that the BMP-7 protein may contribute to apoptosis in the oviduct.

Hu, et al.^55^ (2010) have reported that mesenchymal stromal cell transplantation could increase hematogenesis and reduce apoptosis.

Gashmardi, et al.^56^ (2017) have shown that BMSC transplantation decreased caspase-3 and apoptosis after acute spinal cord injury, showing the role of caspase-3 as an indicator to evaluate treatment efficiency in acute spinal cord injury.

Moreover, the findings in this study agree with El-Haleem, Selim, and Attia^57^ (2018), who have demonstrated that MSC-treated glands show a substantial degree of glandular architecture conservation with abundant CD44-expressing and minimal caspase-3-expressing cells.

Oxidative stress is an imbalance between the production of reactive oxygen species (free radicals) and antioxidant defenses.^58^ Both oxidant and antioxidant substances are controlled by the human body for optimum metabolism, regulation of cellular functions, and cell signaling.^59^ Redox imbalance can damage almost all major cellular components, such as proteins, DNA, and membrane lipids, which may result in cell death.^60^

MDA is a lipid peroxidation product and has been involved as a marker of oxidative status of the organism. Catalase and glutathione are cellular free radical scavengers, which convert oxidant molecules into less reactive ones or make them inactive. Multiple investigations have shown decreased estrogen levels due to an imbalance between oxidant/antioxidant systems after menopause.^61^

This study found significantly higher MDA (the marker of oxidative damage) in the parotid glands of ovariectomized rats in group II than in rats treated with estrogen and BM-MSCs in group III and IV respectively. This indicates that ovariectomy induces lipid peroxidation by systemically increasing oxidative stress. Moreover, these findings are supported by Sánchez-Rodríguez, et al.^62^ (2017), who compared the level of oxidative stress before and after menopause and stated that serum MDA level was significantly increased and serum superoxide dismutase was significantly reduced in postmenopausal women when compared to premenopausal ones. This can produce potential oxidative injury to the cells, leading to the development of numerous ailments such as cardiovascular disease, hypertension, diabetes, vasomotor disturbances, osteoporosis, and depression.

Furthermore, Wei, et al.^63^ (2004) stated that the damage to the parotid ducts is related to the accumulation of reactive oxygen species in their lining cells. Also, Persky, et al.^64^ (2000) explained the increased production of the lipid peroxidation products during menopause by the fact that estrogen can act as an antioxidant because of the similarity in its structure with vitamin E and the phenolic group in its steroid structure. So, its decrease during menopause can increase free radical production.

## Conclusion

The results above show that estrogen deficiency leads to variable degenerative changes in parotid salivary glands of adult female albino rats (with the relative limitation of these changes in the estrogen supplemented group). BM-MSCs and estrogen showed histologically efficacy in regenerating the parotid salivary glands in ovariectomized rats. Hence, BM-MSC therapy can have a promising therapeutic effect on the revival of parotid salivary glands. However, further clinical and histological studies with different parameters should be performed to evaluate the effect of long-term treatment using BM-MSCs as a therapeutic agent. Besides, more complex studies should be done on biological and health consequences of ovariectomized women to test the need of hormonal replacement during menopause with special recommendation to use estrogen and BM-MSCs as a protective measure.

### Limitations of the study

Despite the safety of BMSC therapy, it still needs to be carefully investigated. In addition, further studies are needed to explore the combination of BMSC therapy with estrogen to overcome postmenopausal symptoms, which might be a feasible and efficient therapeutic approach. Although the results of our animal experiments support the potential extrapolation of our hypothesis to human patients, species-specific differences in histology and hormone receptor expression between humans and rats and differences in physiology, genetics, and disease manifestation between both species remain a limiting factor that can restrict the soundness of animal studies as predictors of human outcomes. Furthermore, variations in laboratory procedures, environmental conditions, and the way diseases are induced in animals can complicate translation.
